# PNA-FISH as a new diagnostic method for the determination of clarithromycin resistance of *Helicobacter pylori*

**DOI:** 10.1186/1471-2180-11-101

**Published:** 2011-05-14

**Authors:** Laura Cerqueira, Ricardo M Fernandes, Rui M Ferreira, Fátima Carneiro, Mário Dinis-Ribeiro, Céu Figueiredo, Charles W Keevil, Nuno F Azevedo, Maria J Vieira

**Affiliations:** 1 IBB - Institute for Biotechnology and Bioengineering, Centre of Biological Engineering, Universidade do Minho, Campus de Gualtar 4710-057, Braga, Portugal; 2 IPATIMUP - Institute of Molecular Pathology and Immunology of the University of Porto, Porto, Portugal; 3 Medical Faculty of the University of Porto, Porto, Portugal; 4 Hospital S. João, Department of Pathology, Porto, Portugal; 5 Portuguese Oncology Institute Porto, Department Gastroenterology, Porto, Portugal; 6 Environmental Healthcare Unit, School of Biological Sciences, University of Southampton, Southampton, UK; 7 LEPAE, Department of Chemical Engineering, Faculty of Engineering, University of Porto, Porto, Portugal

## Abstract

**Background:**

Triple therapy is the gold standard treatment for *Helicobacter pylori *eradication from the human stomach, but increased resistance to clarithromycin became the main factor of treatment failure. Until now, fastidious culturing methods are generally the method of choice to assess resistance status. In this study, a new genotypic method to detect clarithromycin resistance in clinical samples, based on fluorescent *in situ *hybridization (FISH) using a set of peptide nucleic acid probes (PNA), is proposed.

**Results:**

The set of probes targeting the point mutations responsible for clarithromycin resistance was applied to *H. pylori *suspensions and showed 100% sensitivity and specificity (95% CI, 79.9-100 and 95% CI, 71.6-100 respectively). This method can also be amenable for application to gastric biopsy samples, as resistance to clarithromycin was also detected when histological slides were tested.

**Conclusions:**

The optimized PNA-FISH based diagnostic method to detect *H. pylori *clarithromycin resistance shown to be a very sensitive and specific method for the detection of clarithromycin resistance in the *H. pylori *smears and also proved to be a reliable method for the diagnosis of this pathogen in clinical samples and an alternative to existing plating methods.

## Background

*Helicobacter pylori *is well known for its ability to colonize human stomach and, while most human carriers are asymptomatic, colonization can lead to the development of several gastric diseases, such as peptic ulcer disease, gastric mucosa-associated lymphoid tissue (MALT) lymphoma and gastric carcinoma.

The over-usage of antimicrobial compounds has led to an increase of *H. pylori *resistance to antibiotics and consequent failure in treatment therapy [1,2]. In accordance with the Maastricht Consensus in Europe, the recommended therapy for *H. pylori *eradication in the stomach mucosa is the use of a proton pump inhibitor associated with two antibiotics, such as metronidazole, amoxicillin or clarithromycin for a 7-14 days period [3]. This therapy, although highly effective, is unselectively proposed to all patients and can imply serious discomfort to patients due to side effects of the antibiotics. Clarithromycin resistance is one of the most prevalent and can reach up to 20% in Southern European countries [1]. The resistance is associated with point mutations in the peptidyltransferase region encoded in domain V of the *H. pylori *23S rRNA gene [2,4,5]. The three most prevalent point mutations are the transitions A2142G and A2143G and the transversion A2142C [1,2,4]. Until now, the antibiotic susceptibility has been detected in clinical laboratories by several phenotypical methods such as the agar dilution method, as recommended by the National Committee for Clinical Laboratory Standards (NCCLS) [6], or the alternative E-test that is considered to be more simple [7-10]. However, these methods are fastidious, time consuming [11], and fail to give any information about the point mutation within the sample [3]. Therefore, molecular methods for the detection of clarithromycin resistance in *H. pylori *have been developed during the last several years in order to overcome these shortcomings. Polymerase chain reaction (PCR) followed by sequencing or reverse hybridization, Real-Time PCR and fluorescence *in situ *hybridization (FISH) with DNA probes are some examples [2,9,12,13]. When compared to PCR-based methods, the FISH technique presents some advantages since it is not so easily affected by DNA contamination, and allows for direct visualization of bacteria in the gastric biopsy specimens [1,2]. Recently, peptide nucleic acid (PNA) probes using FISH have been designed and optimized for the detection of several bacteria, such as *Enterobacter sakazakii*, *Pseudomonas aeruginosa *and *Eschericia coli *[14,15]. PNA molecules are DNA mimics that have the negatively charged sugar-phosphate backbone replaced by an achiral, neutral polyamide backbone formed by repetitive N-(2-aminoethyl) glycine units [16,17]. Although PNA lacks pentoses, specific hybridization between the PNA sequences and nucleic acid complementary sequences still occur according to the Watson-Crick rules [18,19]. The neutral PNA molecule characteristic is responsible for a higher thermal stability (high Tm) between PNA/DNA or PNA/RNA bonding, compared with the traditional DNA probes [17]. Due to this high affinity, PNA probes normally have sequences relatively smaller (13-18 nucleotides) than DNA sequences (at least 18 nucleotides). Moreover, the PNA molecules present more resistance to nucleases and proteases than DNA molecules. When PNA probes are attached to a fluorochrome dye, they can be detected by epifluorescence microscopy or flow cytometry using the fluorescence *in situ *hybridization (FISH) method [16,17,20]. In earlier studies [19], this technique has provided more prompt and robust results in clinical and environmental samples than the traditional culture methods and it has been applied in a wide range of microbiology fields [14,18]. In fact, a PNA-FISH method to determine the presence of *H. pylori *in gastric biopsy specimens has been already developed in our laboratory, using a specific probe (Hp769) [21]. Due to the importance of antibiotic resistance, the aim of this work was to develop and validate a new PNA-FISH based diagnostic method to detect *H. pylori *clarithromycin resistance directly in paraffin embedded gastric biopsies.

## Methods

### Bacterial strains and growth conditions

Thirty three *H. pylori *strains (31 clinical isolates and 2 collection strains), that had their clarithromycin resistance profile determined in this study by sequencing and E-test (see method description below), were used. All strains were maintained on Columbia Agar Base (Liofilchem s.r.l., Roseto D.A., Italy) supplemented with 5% (vol/vol) defibrinated horse blood (Probiológica, Belas, Portugal). Single colonies were streaked onto fresh media every 2 or 3 days, and the plates were incubated in a CO_2 _incubator (HERAcell 150^®^; Thermo Electron Corporation, Waltham, MA, USA) set to 10% CO_2 _and 5% O_2_, at 37°C [21,22].

### Design of PNA oligonucleotide probes for the detection of clarithromycin resistance

PNA probes were designed by adapting the already existing DNA probes, targeting the region of the point mutations described for this antibiotic in *H. pylori *[2]. Since PNA probes usually present higher melting temperatures it was possible to design shorter sequences with 15 nucleotides. The selected probes were Hp1 (A2143G) 5'-GGG TCT CTC CGT CTT-3', Hp2 (A2142G) 5'-GGG TCT TCC CGT CTT-3' and Hp3 (A2142C) 5'-GGG TCT TGC CGT CTT-3'. An additional probe to detect wild type strains (Hpwt 5'-GGG TCT TTC CGT CTT-3') was also included. Afterwards, the selected sequences were synthesized (Panagene, Daejeon, South Korea). The N terminus of the Hp1, Hp2 and Hp3 oligomers was connected to Alexa Fluor 488, and that of the Hpwt connected to Alexa Fluor 594, all via a double AminoEthoxyEthoxy Acetyl linker.

### Fluorescence *in situ *hybridization

As a starting point for the optimization of hybridization conditions the protocol previously described was used [14,21]. Since the different probes only differed in one nucleobase, and for multiplex purposes, a common hybridization temperature was expected for all probes. Based on the brightest signals and specificity of the results, the best performance was obtained at 70°C (data not shown). *H. pylori *sensitive or resistant strain suspensions were prepared in water and 20 μl of each suspension was dispensed in 8 mm well slides (Marienfeld, Lauda-Königshofen, Germany) and then allowed to air dry. For permeabilization and fixation of bacteria, 30 μl of 4% paraformaldehyde (wt/vol) were placed in the wells with care to cover the entire surface, followed by 50% (vol/vol) ethanol for 10 minutes each, and then allowed to air dry. Approximately 20 μl of hybridization solution containing a mixture of the four probes were added to the fixed smears, which were then covered with coverslips and incubated for 1 hour at 70°C. Each 1 ml of hybridization solution contained 200 nM of the probes mixture, 10% (wt/vol) dextran sulphate, 10 mM NaCl, 30% (v/v) formamide, 0.1% (wt/vol) sodium pyrophosphate, 0.2% (wt/vol) polyvinylpyrrolidone, 0.2% (wt/vol) FICOLL, 5 mM disodium EDTA, 0.1% (vol/vol) Triton X-100 and 50 mM Tris-HCl (all from Sigma-Aldrich, Sintra, Portugal, except disodium EDTA that was from Pronalab, Lisbon, Portugal). Subsequently, the slides were transferred to a Coplin jar containing prewarmed (70°C) washing solution, that consisted of 5 mM Tris Base, 15 mM NaCl and 1% (vol/vol) Triton X-100 (all from Sigma-Aldrich, Sintra, Portugal), where the coverslips were carefully removed. The washing step was carried out for 30 minutes at 70°C. The slides were allowed to air dry and mounted with one drop of mounting oil and covered with a coverslip.

### Specificity and sensitivity of PNA probes

After optimizing hybridization conditions, experiments with the PNA-FISH were performed on the 33 available strains in order to confirm the practical specificity and sensitivity of the probes. These results were compared with the gold standard susceptibility culturing test (E-test) and with the presence/absence of mutations in the 23S rRNA gene.

### Validation of the testing protocol in gastric biopsy slides for clinical application

To validate the method in the stomach tissue, thirty nine paraffin-embedded gastric biopsy specimens from patients with known resistance antibiotic profile by antibiogram were used. The study was in accordance with the institutional ethical standards. Informed consent was obtained from the patients. Three-micrometer thick paraffin cuts were deparaffinized and rehydrated in xylol and ethanol based on a protocol previously described [21]. Sections were emerged in xylol (Fisher Chemical, Leicestershire, U.K.) three times (firstly for 15 minutes, and then twice for 10 minutes each), absolute ethanol (Panreac, Barcelona, Spain) (twice for 7.5 minutes each) and ethanol decreasing concentrations (95%, twice for 7.5 minutes each; 80%, 10 minutes; 70%, 10 minutes; 50%, twice for 15 minutes each). Finally sections were immersed in 1% (vol/vol) Triton X-100 (Sigma-Aldrich, Sintra, Portugal) solution for 20 minutes at 70°C. Histological slides were then allowed to air dry and the hybridization protocol previously described for smears, with the exclusion of the fixation step, was used. The completion of the whole procedure takes 4 h 15 m.

### Susceptibly test: E-test

In order to confirm the susceptibility profile, the minimal inhibitory concentration (MIC) of each strain was determined by the E-test, in accordance with the company instructions (AB Biodisk, Biomérieux, Portugal). Briefly, 2 day-old pure cultures were inoculated into Mueller-Hinton broth, supplemented with 5% (vol/vol) fetal calf serum [23] and the turbidity of the inoculum adjusted to McFarland standard 3 [7]. Agar plates containing Mueller-Hinton supplemented with 5% (vol/vol) defibrinated horse blood (Probiológica, Belas, Portugal) were inoculated by swabbing the surface with the inocula. One E-test strip was applied on the surface of the plate, after drying. The plates were incubated in a CO_2 _incubator (HERAcell 150^®^; Thermo Electron Corporation, Waltham, MA, USA) set to 10% CO_2 _and 5% O_2 _at 37°C for 72 h or until visible inhibition ellipse was seen [2,7,23]. Strains were considered susceptible when the MIC was < 1 μg/ml, and resistant when the MIC was > 1 μg/ml [9].

### Assessment of clarithromycin resistance in gastric tissues by PCR and sequencing

Total DNA was extracted from biopsy samples after digestion with Proteinase K for at least 12 hours at 55°C. Proteinase K was inactivated by incubation at 95°C for 10 minutes. Ten microliters of the lysates were used for PCR amplification of *H. pylori *23S rRNA gene as previously described [24]. PCR products were sequenced using BigDye Terminator v3.1 Cycle Sequencing Kits (Applied Biosystems, CA, USA) and run in an ABI Prism 3130 DNA automated sequencer (Applied Biosystems). In some *H. pylori *isolates, PCR and sequencing were used to characterize the 23S rRNA gene.

### Microscopic visualization

Visualization of samples never exceeded 48 h after the experimental procedure. Smears or histological slides were observed using an epifluorescence microscope (BX51 Olympus, Hamburg, Germany) equipped with filters adapted to the Alexa Fluor (488 and 594) signalling molecules within the probes. The filters that were not sensitive for the reporter molecules were used as negative control.

## Results and Discussion

### Specificity and sensitivity of the PNA-FISH probes

In order to confirm the practical specificity and sensitivity of the probes, PNA-FISH was performed on the 33 available strains (table 1). The original genotyping of the strains was confirmed by sequencing, and 20 isolates were identified as clarithromycin resistant. Of these, 10 presented the A2143G mutation, eight the A2142G mutation and one the A2142C mutation. In one case, different genotypes in the same strain (WT and A2143G) were observed, and this strain was considered resistant. The comparison between PNA-FISH and sequencing showed a correlation of 100%.

**Table 1:** PCR, E-test and FISH results of the detection of clarithromycin resistance in *H. pylori *clinical isolates

Strain	Genotype	PCR	E-test	PNA-FISH
3939^1^	WT	S	S	S
2277^1^	WT	S	R	S
2406^1^	WT	S	S	S
2424^1^	WT	S	S	S
3183^1^	WT	S	S	S
3131^1^	WT	S	S	S
3148^1^	WT	S	S	S
2452A^1^	WT	S	S	S
2448^1^	WT	S	S	S
2708^1^	A2142G	R	R	R
2712^1^	A2142G	R	R	R
3941^1^	WT/A2143G	R	R	R
2191^1^	A2143G	R	R	R
2009^1^	A2143G	R	R	R
1162^1^	A2143G	R	R	R
1987^1^	A2143G	R	R	R
2053^1^	A2143G	R	R	R
2538^1^	A2143G	R	R	R
2539^1^	A2143G	R	R	R
2768^1^	A2143G	R	R	R
166^2^	A2142C	R	R	R
167^2^	A2143G	R	R	R
168^2^	A2142G	R	R	R
169^2^	WT	S	S	S
7.83^3^	A2143G	R	R	R
7.11^3^	A2142G	R	R	R
7.38^3^	A2142G	R	R	R
6271^3^	A2142G	R	R	R
7.36^3^	A2142G	R	R	R
6231^3^	A2142G	R	R	R
968^1^	WT	S	S	S
NCTC 11637^4^	WT	S	S	S
ATCC 700392^4^	WT	S	S	S

Some strains were also sequenced in our labs to guarantee that no contamination had occurred during culture maintenance

^1 ^Dr. M. Oleastro (National Institute of Health, Lisbon, Portugal); ^2^Dr. R. Haas (Max von Pettenkofer Institute for Hygiene and Medical Microbiology, Ludwig Maximilians University of Munich, Germany); ^3^Dr. G. Perez-Perez (NYU Langone Medical Center, New York, USA); ^4 ^Type strain; R: Clarithromycin resistant (MIC > 1 μg/ml); S: Clarithromycin susceptible (MIC < 1 μg/ml); WT: Wild Type.

There are other less prevalent point mutations referred in the literature [25-28], but are surrounded by controversy since their association to clarithromycin resistance have not been definitely proved [1,29]. In addition to that, some reports presented clarithromycin resistance mechanisms other than point mutations, such as efflux pumps or rRNA methylation [30] that can be revealed with phenotypic methods, although they are not detected by genotypic methods that are specific to certain cellular events as is the case of the probes here described. In the present manuscript, one of the strains tested gave different results between E-test (MIC 32 μg/ml) and PNA-FISH (only hybridized with the Hpwt) showing 95.5% of similarity between the two methods (table 2). This apparently discrepant observation may be attributed to the presence of other 23S rRNA gene mutations known to confer phenotypic resistance or, alternatively, to additional mechanisms of resistance. Despite this decrease in sensitivity, it is known that the three mutations referred to in this study were revealed to be the more frequently associated with macrolide resistance. De Francesco and co-workers [30] stated that more than 90% of primary clarithromycin resistance strains from western countries are related with A2142G, A2142C and A2143G mutations.

**Table 2:** Comparison between PNA-FISH methodology, PCR-sequencing and E-test for detection of clarithromycin resistance in 33 *H. pylori *strains

	PNA-FISH	
	
	Resistant	Susceptible
E-test		
Resistant (21)	20	1
Susceptible (12)	0	12

PCR-sequencing		
Resistant (20)	20	0
Susceptible (13)	0	13

**Figure 1:**
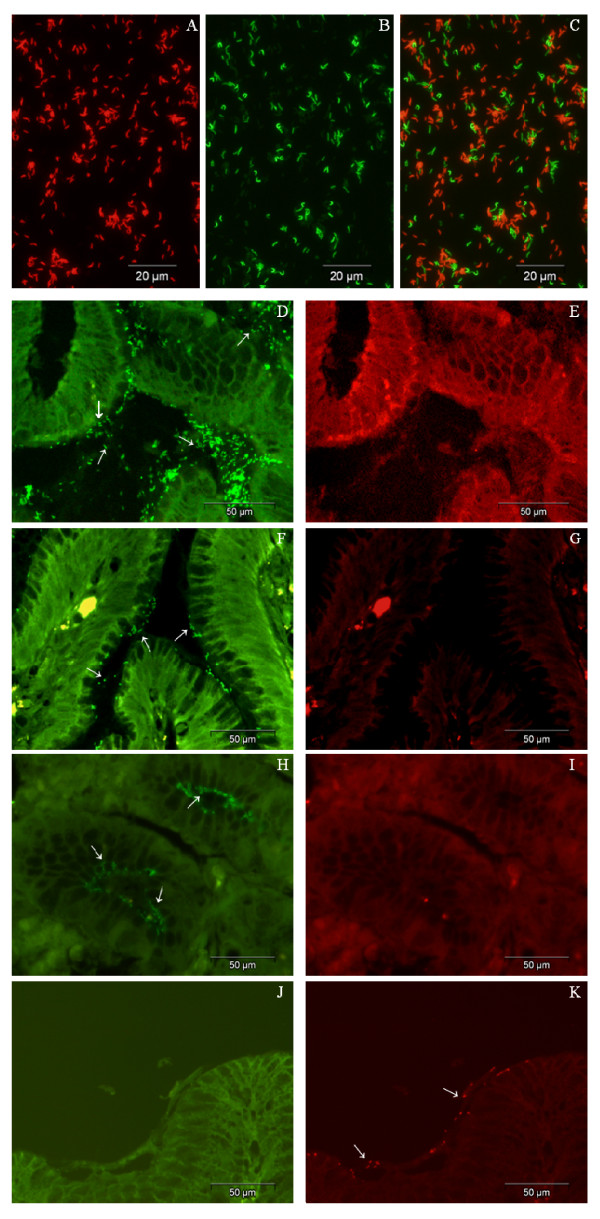
**PNA-FISH detection**. A)-C) In smears: A) Susceptible strain in the red channel; B) Resistant strain in the same microscopic field in the green channel; C) Superimposition of both channels. D)-K) In gastric biopsy histological slides. D) Strain visualization using the Hp1 (A2143G) PNA probe; F) Hp2 (A2142G) PNA probe; H) Hp3 (A2142C) PNA probe; K) Hpwt (wild type strain) PNA probe; E),G),I) Visualization of the same microscopic field of D),F),H) with the red channel (negative controls for Hp1, Hp2 and Hp3); J) Visualization of the same microscopic field of K) with the green channel (negative control for Hpwt). Arrows indicate the presence of *H. pylori *infecting the gastric mucosa. (Original magnification × 600).

From the three mutations, the one that is less frequent is the A2142C transversion [1,12], and in this study we were only able to test one strain with that mutation. Nevertheless, the available strain was always detected when the Hp3 probe was present in the hybridization solution. Probes Hp1, Hp2, and Hp3, hybridized only with the resistant strains that had the corresponding point mutations conferring clarithromycin resistance and as such presented 100% sensitivity (95% CI, 79.9-100) and 100% specificity (95% CI, 71.6-100). The set of probes can discriminate the resistant and susceptible strains, even though they only have one mismatch. We next further tested the method using a mixture of the four probes simultaneously in a multiplex detection (figure 1; A-C). In this case, the detection of point mutations was even more robust, which is possibly due to the fact that all probes target the same locus, and as such there is a competition effect between them. However, with the mixture it is only possible to discriminate between clarithromycin resistant and clarithromycin sensitive strains, as opposed to the discrimination between point mutations that was conferred by using the probes separately. In practical terms and considering the application of the PNA-FISH to the clinical setting, the mixture of probes introduces an important simplification to the method.

### Validation of the testing protocol in gastric biopsy slides for clinical application

Considering the application of the PNA-FISH method in clinical settings, we used the developed PNA probes to identify and differentiate clarithromycin resistant and susceptible *H. pylori *strains in histological slides of gastric biopsy samples. Results clearly show that it is possible to discriminate susceptible from resistant *H. pylori *strains and, in the latter group, to detect the three different mutations, using fluorescence microscopy (figure 1; D-K). Taking into consideration the antibiogram as the gold standard, the PNA-FISH method showed specificity and sensitivity of 90.9% (95% CI, 57.1-99.5) and 84.2% (95% CI, 59.5-95.8), respectively (data not shown). These can probably be explained by the existence of another mechanism of resistance apart from the three point mutations assessed in this study. In fact, association between A2142C, A2142G and A2143G mutations and clarithromycin resistance was defined as approximately 84% in a world wide data compilation [3].

Although, these results need to be confirmed in a larger series of cases, they indicate that PNA-FISH is applicable to clinical samples and may be useful for the selection of the most adequate antibiotic combination to be used for *H. pylori *eradication. Moreover, this method is simple to perform and the procedure is fast (4 h 15 m), indicating that results can be provided to clinicians simultaneously with the histological diagnosis.

## Conclusions

Resistance to antibiotics, namely to clarithromycin, is one of the causes of treatment failure in *H. pylori *eradication [1]. For this reason, it is the most beneficial to detect resistance to clarithromycin prior to antibiotic therapy. Standard culturing methods (E-test, agar dilution) have been used for this purpose, despite several shortcomings: these methods are time consuming and *H. pylori *is difficult to grow in culture; there is the risk of contamination of samples during transportation leading to overgrowth of other bacteria that may mask the growth of *H. pylori*; these methods do not provide any information regarding the specific point mutation(s) in each resistant strain [12]. Other alternative molecular based methods require DNA extraction followed by PCR amplification and sequencing for the identification of the mutation(s) [4,9,13].

Herein we describe the applicability of PNA-FISH methodology to clinical material, namely gastric biopsy samples [2,21], thus overcoming the need of culturing steps and/or PCR/sequencing procedures and enabling rapid initiation of appropriate antibiotic therapy until culture confirmation can be obtained several days later [1]. Furthermore, the required equipment, a fluorescent microscope equipped with adequate filters for fluorochromes, is easy to handle for routine diagnostic purposes. For centres using routine cultures of *H. pylori*, the complementary use of PNA-FISH methodology to smears of bacteria will increase the sensitivity of the detection of resistant strains in clinical samples.

## Competing interests

Authors LC, NFA and MJV are inventors on a patent application describing the four PNA probes reported here (PT PAT 40801-09). This is currently held by University of Minho (UM) which is a current employer of LC and MJV and a previous employer of NFA. All the other authors are aware of the patent, agreed with its submission and do not present any competing interest.

## Authors' contributions

LC conceived of the study and participated in its design and drafted the manuscript. Carried out the PNA probes design, PNA-FISH, E-test and PCR-sequencing assays. RMF participated in the PNA-FISH assays and in the design of the study. RMF carried out the PCR-sequencing studies. FC participated in the design of the study and helped to draft the manuscript. MDR participated in the design of the study and helped to draft the manuscript. Provided the gastric samples for the study. CF participated in the design of the study, on the PCR-sequencing analysis, and helped to draft the manuscript. CWK participated in the design of the study and helped to draft the manuscript. NFA conceived of the study and participated in its design and coordination and helped to draft the manuscript. MJV conceived of the study and participated in its design and coordination and helped to draft the manuscript. All authors read and approved the final manuscript.
